# Supplementary material for: The rs16969968 Tobacco Smoking-Related Single-Nucleotide Variant Is Associated with Clinical Markers in Patients with Severe COVID-19
Source: Int J Mol Sci. 2023 Jun 6;24(12):9811. doi: 10.3390/ijms24129811 (PMC10298344; doi:10.3390/ijms24129811)
Supplement: Supplementary file 1 [file ijms-24-09811-s001.zip › ijms-2410126-supplementary.pdf]

Table S1. Clinical laboratory tests at 7-10 days of hospitalization

Variable	*GA+AA*	*GG*	*p*
WBC (x10^3/µL)	9.7 (7.2 - 13.3)	10.3 (8.1 - 13.9)	0.131
Lymphocytes (x10^3/µL)	0.8 (0.4 - 1.1)	0.8 (0.5 - 1.3)	0.467
Platelets (x10^3/µL)	257 (190 - 369)	282 (204 - 360)	0.198
LDH (UI/L)	315 (238 - 365)	325 (265 - 402)	0.270
D-Dimer (µg/mL)	1.79 (1.00 - 3.22)	1.68 (0.92 - 3.16)	0.671
ESR (mm/h)	28 (26 - 30)	28 (22 - 36)	0.900
CRP (mg/dL)	7.6 (2.6 - 13.4)	6.9 (2.5 - 13.8)	0.983
Fibrinogen (mg/dL)	593 (495 - 693)	609 (511 - 721)	0.269
Procalcitonin (ng/mL)	0.15 (0.09 - 0.72)	0.13 (0.06 - 0.48)	0.137
Ferritin (ng/mL)	897 (592 - 1677)	936 (557 - 1364)	0.503

Showing medians and interquartile ranges (p25 - p75). The p-value was obtained using the Mann-Whitney U test.

WBC: white blood cells. LDH: lactic dehydrogenase. ESR: erythrocyte sedimentation rate. CRP: C-reactive protein.

Table S2. Intra-group comparison of clinical laboratory tests at the admission and during hospitalization (7-10 days).

Variables	GA+AA (n= 115)		GG (n= 414)	
	Admission	Hospitalization	Admission	Hospitalization
WBC x10³/μL	9.8 (8.3- 13.2)	9.7 (7.2- 13.3)	10.4 (8.0- 13.5)	10.3 (8.1- 13.9)
Lymphocyte x10³/μL	**2.0*** (0.7- 6.3)	**0.8*** (0.4- 1.1)	**2.1**** (0.7- 6.9)	**0.8**** (0.5- 1.3)
Platelets x10³/μL	261 (213- 327)	257 (190- 369)	**258**** (189- 334)	**282**** (204- 360)
LDH (UI/L)	**393*** (290- 490)	**315*** (238- 365)	**387**** (301- 508)	**325**** (265- 402)
D Dimer (μg/mL)	1.41 (0.77- 3.26)	1.79 (1.00- 3.22)	1.67 (0.60- 3.77)	1.68 (0.92- 3.16)
CRP (mg/dL)	**12.0*** (5.8- 19.5)	**7.6*** (2.6- 13.4)	**12.3**** (6.6- 20.9)	**6.9**** (2.5- 13.8)
Fibrinogen (mg/dL)	**707*** (575- 781)	**593*** (495- 693)	**666**** (569- 778)	**609**** (511- 721)
Procalcitonin (ng/mL)	0.24 (0.08- 0.72)	0.15 (0.09- 0.72)	**0.21**** (0.09- 0.65)	**0.13**** (0.06- 0.48)
Ferritin (ng/mL)	**990*** (669- 2368)	**897*** (592- 1677)	1064 (637- 1892)	936 (557- 1364)

*Statistically significant difference in GA+AA at admission vs. 7-10 days of hospitalization ** Statically significant difference in GG at admission vs. 7-10 days in the hospital. WBC: White blood cell, LDH: lactic dehydrogenase, CRP: C-reactive protein. Statistically significant values are shown in bold.

Figure S1. rs16969968 in *CHRNA5*

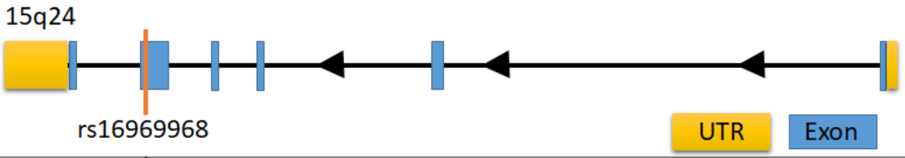

Description	rs16969968	
Gene level [35]	G	A
Protein level [35]	Aspartic acid	Asparagine
Functional studies en nicotine dependence [36]	Promote Ca^{2+} permeability	Might inhibit the Ca^{2+} permeability
In the brain [37, 38]	The presence of the A allele shows low mRNA expression of CHRNA5 in comparison to the carriers of allele G	
Functional consequences in COPD patients [39]	The number of primary cilia is increased in the bronchial epithelium and decreased in the bronchiolar epithelium of patients carrier of A allele	
